# Reading of ingroup politicians’ smiles triggers smiling in the corner of one’s eyes

**DOI:** 10.1371/journal.pone.0290590

**Published:** 2024-04-18

**Authors:** Edita Fino, Michela Menegatti, Alessio Avenanti, Monica Rubini

**Affiliations:** 1 Department of Psychology “Renzo Canestrari”, Alma Mater Studiorum Università di Bologna, Bologna, Italy; 2 Centro Studi e Ricerche in Neuroscienze Cognitive, Department of Psychology “Renzo Canestrari”, Alma Mater Studiorum Università di Bologna, Campus di Cesena, Cesena, Italy; 3 Centro de Investigación en Neuropsicología y Neurociencias Cognitivas, Universidad Católica del Maule, Talca, Chile; Yeditepe University, TURKEY

## Abstract

Spontaneous smiles in response to politicians can serve as an implicit barometer for gauging electorate preferences. However, it is unclear whether a subtle Duchenne smile–an authentic expression involving the coactivation of the zygomaticus major (ZM) and orbicularis oculi (OO) muscles–would be elicited while reading about a favored politician smiling, indicating a more positive disposition and political endorsement. From an embodied simulation perspective, we investigated whether written descriptions of a politician’s smile would trigger morphologically different smiles in readers depending on shared or opposing political orientation. In a controlled reading task in the laboratory, participants were presented with subject-verb phrases describing left and right-wing politicians smiling or frowning. Concurrently, their facial muscular reactions were measured via electromyography (EMG) recording at three facial muscles: the ZM and OO, coactive during Duchenne smiles, and the corrugator supercilii (CS) involved in frowning. We found that participants responded with a Duchenne smile detected at the ZM and OO facial muscles when exposed to portrayals of smiling politicians of same political orientation and reported more positive emotions towards these latter. In contrast, when reading about outgroup politicians smiling, there was a weaker activation of the ZM muscle and no activation of the OO muscle, suggesting a weak non-Duchenne smile, while emotions reported towards outgroup politicians were significantly more negative. Also, a more enhanced frown response in the CS was found for ingroup compared to outgroup politicians’ frown expressions. Present findings suggest that a politician’s smile may go a long way to influence electorates through both non-verbal and verbal pathways. They add another layer to our understanding of how language and social information shape embodied effects in a highly nuanced manner. Implications for verbal communication in the political context are discussed.

## 1. Introduction

In the political arena, people strive to understand how to capture political support [1]. At the nonverbal level, spontaneous facial expressions such as a fleeting smile detected in an unaware observer’s face, can be used to index affective reactions to political candidates and to reliably gauge electorate preference [2]. An indication of positive affect in a smile can be inferred by the contraction of both the lip corner muscle (i.e., the zygomaticus major, ZM), which pulls the lips up into the familiar curve we most commonly identify with smiling, and the orbicularis oculi (OO), which creates wrinkles around the outer corner of the eyes, commonly referred to as the Duchenne marker [3, 4].

Relevant to the present study is the evidence that unintended smile reactions are similarly triggered when seeing other people smiling, hearing their laughter, or reading descriptions of their smiling in written media [5–9]. In line with sensorimotor simulation accounts of facial expression recognition, this body of research indicates that as we perceive another person’s smile, we partially simulate or reproduce the smile in our sensorimotor system [10]. Albeit automatic, facial effects triggered by perception of others’ expressions are modulated by social factors such as the similarity between observer and target, positive attitudes, social context and group membership [11, 12]. Along these lines, the present study investigates a relatively neglected trigger of smiles, namely that of linguistic portrayals of politicians smiling. We employed facial electromyography (EMG) to detect smile responses during exposure to verbal depictions of political leaders smiling and frowning and examined whether different smiling patterns would emerge in faces of voters for ingroup but not outgroup politicians’ smiles reflecting positive affect and likely political support.

### 1.1 Smiles in the political context

Smiles are one of the main channels through which political leaders tend to induce feelings of happiness, confidence, and reassurance in others to the aim of increasing positive evaluations, obtaining consensus and expanding electoral support [13, 14]. However, far from being simple readouts of positive affect, smiles have been shown to signal a wide range of socially relevant information and to regulate social interactions in a highly nuanced fashion [10, 15]. Indeed, the political context offers one of the most ecologically valid settings for the smiles’ nuanced meanings to emerge [17]. For instance, in addition to genuine enjoyment, smiles often serve as signals of a willingness to cooperate and absence of threat (i.e., affiliative smiles). They may also be employed to express feelings of superiority and dominance towards a fierce opponent (i.e., dominance smiles), indicating in this case more negative rather than positive affect [15–19].

Smiles convey complex social information through the combined activation of a variety of facial muscles [20]. For instance, when the contraction of the ZM which is commonly identified with a smile, is coupled with the contraction of the OO (the eye corner muscle responsible for creating small wrinkles around the eyes), then the smile is commonly perceived to express positive affect [3, 4]. Hence, a smile that extends to a person’s eyes, is deemed to signify genuine enjoyment and authenticity [4, 15, 16, 21] compared to smiles activating the lip corner muscle only (ZM), which may often reflect social expressions of politeness and may at times be also associated with negative affect [22]. This distinction is especially relevant if smiles are displayed spontaneously, occurring outside one’s conscious control, as research suggests that when smiles are produced deliberately, the contraction of the eye corner muscle (i.e., Duchenne marker) may not always correlate with genuine positive feelings [23]. However, spontaneous or not, there is consensus in the literature that Duchenne smiles lead to more favorable interpersonal perceptions. Indeed, people producing Duchenne smiles are rated more positively (for a meta-analysis see [24]), and they are facially responded to more than when producing non-Duchenne smiles [25–27].

### 1.2 Sensorimotor simulations accounts of facial effects

Research shows that smiles can be automatically triggered not only by visual and auditory cues of smiling expressions [7, 9], but also by written descriptions of someone smiling [6, 28–31]. Proponents of sensorimotor simulation accounts hold that as we perceive another person’s smile, we engage in a partial simulation or reproduction of the smile in our sensorimotor system. This process involves the reactivation of related concepts, feelings and autonomic and behavioral changes (for a review see [32]) and enhances one’s capacity to respond emphatically [33] and recognize others’ emotions based on nuanced meanings of their facial expressions [15, 34–37]. Such claims are supported by evidence showing that when people are blocked in their ability to simulate smiles with their facial muscles [30, 36, 38], or when their face representation in the sensorimotor cortex is disrupted by brain stimulation [39–41], their ability to correctly identify or judge emotional expressions of others is significantly impaired.

### 1.3 Social modulation of unintended facial effects

Although corresponding facial expressions are very rapidly evoked in perceivers’ facial muscles (within 500 ms), representing a largely automatic stimulus-induced effect, they are not impervious to top-down modulation by social information. For instance, conceptual knowledge about the person doing the smiling and the social context in which the smile is displayed can implicitly affect observer’s expectations or inferences about the meaning expressed by smiles, reactivating related feelings and emotions accordingly, thus influencing whether and to what extent smiles will be corresponded [42, 43]. Indeed, research has demonstrated rapid modulation by social information of such facial effects, suggesting a facilitation for people we favor and members of our own social group, and a reduction or at times suppression of the effect for people we dislike and outgroup members (for reviews, see [12, 44]).

While there is ample behavioral and neurophysiological evidence supporting stronger sensorimotor responding to ingroup than to outgroup members across a range of tasks, social groups and contexts [45–50], only a few studies have examined facial reactions to smiles in the political intergroup context. Early research employing electromyography (EMG) to measure facial responses to politicians’ smiles [2, 51] has mainly relied on naturalistic stimuli such as short videoclips of politicians smiling. For instance, McHugo & Lanzetta [2] presented participants with 30-second-long video excerpts of politicians smiling and found that the ZM response was more enhanced among supporters than opponents of the Republican candidate Regan. These results were contrasted by a later adaptation of that study by Bourgeois and Hess [51], who recorded facial activity at the ZM and OO while participants were exposed to 13s video sequences of smiling conservative and liberal political leaders. They found that both ingroup and outgroup politicians’ smiles induced the same level of facial muscle activation among supporters of the conservative political leader.

A more recent study [29] adopted a novel perspective by examining linguistic portrayals of left and right-wing politicians’ emotion expressions. This study employed EMG recording from the ZM and the CS facial muscles, which are involved in smiling and frowning, respectively, in left and right-wing votes while they were reading about ingroup and outgroup politicians’ expressions. Findings revealed a more enhanced smile amongst right-wing participants while reading of ingroup compared to outgroup politicians’ portrayals of smiles (in line with results of McHugo & Lanzetta [2]). In contrast, left-wing participants exhibited a similar facial response to ingroup and outgroup politicians’ smiles aligning more with results of Bourgeois & Hess [51]. Additionally, left-wing participants showed a higher ZM activity in response to outgroup politicians’ frowns, an indication of a “*schadenfreude*” effect, or pleasure derived by someone’s misfortune. This study holds significance because it demonstrated that politicians’ smiles can induce smiles even when they are presented conceptually, that is through written media, extending previous research based on naturalistic stimuli. The use of linguistic material allows to control for the natural variability in the target politician’s smile type and intensity, which is inherent in research employing naturalistic stimuli [2, 51] and may independently affect observers’ behavior [13]. The study of Fino et al. [29] demonstrated the real-world applicability of facial reactions to linguistic portrayals of political candidates in predicting electoral outcomes. Notably, the favorable response detected in the smile reaction of right-wing participants to the left-wing political leader rising into power Matteo Renzi foreshadowed Renzi’s subsequent electoral support from a broad electorate base including both left-and right-wing voters in the ensuing national elections in Italy. However, a limitation of this study concerns the over reliance on ZM activity as the only index of readers’ smiling reaction. Such one-size-fits-all approach to smile reactions did not allow for disambiguation of more nuanced smiling patterns amongst supporters and opponents of target politicians. For instance, it cannot be ruled out that beyond a similar ZM activation, left-wing participants may have been reacting with different kind of smiles altogether, reserving a more malicious one (i.e., dominant smile) for outgroup politicians as suggested by their *schadenfreude* response.

### 1.4 The present study

Current evidence about facial reactions to politicians’ smiles is mixed, with some studies [2, 29] reporting that smiles are enhanced for ingroup compared to outgroup leaders (for right-wing participants), and other studies [29, 51] showing a similar response across political groups (for left-wing participants). This begs the question of whether there may be more fine-grained differences underlying such effects. Prior work has employed different experimental procedures and was focused mainly on the ZM activation (except for [51]). The question of whether the activation of the OO–a key facial muscle involved in smiles signaling positive affect (otherwise known as Duchenne smiles)–could further qualify facial responses to politicians’ smiles, depending on political affiliations, deserves further attention. To date, no study has examined whether the Duchenne marker, indicating positive affect in a smile, can be observed when participants are facially responding to linguistic representations of others’ smiling, as most research on Duchenne smiles has traditionally relied on visual stimuli. The present work addresses these gaps by investigating whether reading of ingroup or outgroup politicians’ portrayals of smiles induces qualitatively different smile reactions among readers of same and opposing political orientation, reflecting enjoyment for ingroup but not outgroup politician’s smile depictions. Specifically, we measured unintended facial reactions to linguistically presented stimuli. Moreover, by indexing facial responses across different time windows in a 0–3s post-stimulus onset interval, this is also the first study to explore automatic facial reactions to verbal descriptions of politicians’ smiles as they unfold through time.

In a laboratory reading task, participants were exposed to verbal phrases describing ingroup and outgroup politicians smiling and frowning, while their spontaneous facial reactions were measured via EMG from the ZM, OO and the CS. Based on the evidence reviewed above, showing mixed results in terms of ZM discriminating between ingroup and outgroup smiles, we expected that facial responses to phrases depicting smiling ingroup politicians would involve a significant activation in both the lip puller (ZM) and eye corner (OO) facial muscles, indicating positive affect, which would be further corroborated by higher levels of positive emotions reported for ingroup compared to outgroup politicians. In response to outgroup politicians’ smiles, we expected to find lesser or similar activation of the ZM and no activation of the OO, as well as higher levels of negative emotions reported towards these later. In addition, we expected a more enhanced frown response (CS) for ingroup compared to outgroup politicians’ frown expressions.

## 2. Materials and methods

### 2.1 Participants

Thirty undergraduate students at the University of Bologna (27 females, mean age 22 years old) participated in the study for academic credit. Data were gathered from May to October 2013 at the University of Bologna. Participants were selected from a pool of 120 students filling out a political identification scale from 1 (left) to 7 (right). Those agreeing to participate in the present study reported being left-wing and scored below 3 on the political identification scale. All subjects had normal or corrected-to-normal vision, were right-handed and were naïve to the purpose of the experiment. The study was not preregistered, the protocol was approved by the Bioethics committee of the University of Bologna and was carried out in accordance with the ethical standards of the 2013 Declaration of Helsinki [52]. Prior to the start of the experiment participants read the ethical approval statement and provided written informed consent. Data from one subject were excluded from final analysis due to technical problems with electrode adherence.

### 2.2 Stimuli and procedure

#### 2.2.1 General procedure and behavioral task

To conceal the real purpose of the experiment, participants were told they would participate in a study concerning the language of politics and that we were interested in how specific phrases describing politicians were evaluated. As a cover story for electrode placement in the face they were told that skin conductance would be measured as a psychological index of arousal during the reading task. Participants were assessed individually in a lab where they were first prepared for electrode placement and were informed about the (masked) objective of the study. Then they were invited to sit in front of a computer for the completion of a reading task consisting in sequential presentation of a series of subject-verb sentences on a monitor. Participants sat approximately 60 cm from a 19-inch computer monitor. They were instructed to read each target sentence sequentially presented on the center of the monitor and rate them on a 5-point liking scale (1 = I don’t like it at all; 5 = I like it very much). Each sentence was preceded by a fixation cross lasting 1s, which was followed by the target phrase remaining on the screen for 3 s and followed by the liking scale that disappeared upon response (mean RT was ~ 4 s). Then the screen remained blank for 1s in the inter-trial interval. Thus, the inter-stimulus interval was ~10 s allowing sufficient time for facial muscles to relax after stimulus presentation.

The stimulus material consisted in 6 Italian action verbs (3 positive and 3 negative facial expressions) and 3 neutral fillers taken from Fino et al. [29]. Positive verbs included action verbs referring directly to facial expressions: *to smile* (sorridere), *to laugh* (ridere), (riddachiare); Similarly, negative verbs included action verbs: *to frown* (corrucciare), *to grin* (aggrotare), (accigliare). Fillers included: *to work* (lavorare); to walk (camminare). All verbs were matched for word length and frequency (see masked). Verbs were presented in the present tense and were embedded into subject-verb sentences with each target verb being attributed to either a left-or right-wing politician (e.g., ‘*Bersani smiles*’, ‘*Alfano frowns’*). Based on a pilot study [masked], we selected two left-wing politicians (Bersani and Renzi) and two right-wing politicians (Alfano and Berlusconi) from the main political parties (Democratic party and People of Freedom, respectively) at the time of the study. For each participant 3 blocks of stimuli were presented with each block containing all the 6 facial expression verbs and 3 neutral verbs attributed to 4 left-and right-wing politicians, resulting in 36 stimuli presentations per block, and a total of 108 trials altogether. Prior to the start of the experiment, 10 practice trials were administered for participants to familiarize themselves with the computer task. During the experiment, the verbal stimuli were presented in a random order with E-prime software [53].

After the task, participants completed a questionnaire including manipulation checks and assessing their attitudes on a multiplicity of measures. They were first asked to indicate their political orientation on the same item used prior to the experiment and report on the valence of each stimulus sentence (see next paragraph). Then they indicated the political affiliation of each politician by choosing between two options (left or right) and rated how much each target politician represented the political wing they had indicated. In the end participants were asked about their ideas regarding the purpose of the experiment, and none of them reported being aware of the study hypotheses or suspected that facial muscular reactions were being measured. Then, they were debriefed and were dismissed.

#### 2.2.2 Apparatus and data acquisition

Facial muscle activity was measured during stimulus presentation using miniature Ag/AgCl surface electrodes (4mm) attached over the left ZM, OO and CS muscles. Site preparation and electrode placement were done following standard procedure guidelines [54, 55]. The skin was cleaned and prepared for electrode placement to reduce skin impedance to less than 10 kΩ. The raw EMG activity was measured with Biopack Systems MP36 data acquisition unit at a sampling rate of 1000 Hz using two bipolar channels and a gain of 1,000. The EMG signal was pass filtered with a 20–250 Hz passband and a 50 Hz notch filter and was full wave rectified offline.

### 2.3 Dependent variables

#### 2.3.1 Manipulation checks

To check participants’ political orientation, they were asked to report on the item: “Where would you locate yourself politically in a continuous scale from 1(*left*) to 7 (*right*)”. To check whether the target politicians were considered as being left- or right-wing, participants were asked how much they represented the right- and the left-wing respectively on a 7-point Likert scale from 1 *(not at all)* to 7 *(very much)*. To check whether positive and negative subject-verb sentences were evaluated as positive and negative respectively, participants were asked to assess the valence of each phrase on a 7-point Likert scale from 1 (*very positive*) to 7 (*very negative*).

#### 2.3.2 Facial muscle EMG activity

Smile reaction patterns were assessed through EMG recording at the ZM and OO muscle sites. Frown reactions were measured by EMG recording of the CS activity. The EMG signal in the time window of interest (1000ms pre-stimulus to 3000ms post-stimulus onset) was rectified and root mean square (RMS) transformed. On each trial, mean EMG amplitude in the post-stimulus period was baseline-corrected using mean EMG amplitude during the pre-stimulus period. Phasic facial EMG responses (in microvolts, mV) were scored across bins of 100ms and averaged over intervals of 500ms during the 3s of stimulus presentation. The EMG signal was visually inspected offline and screened for electrical noise and artifacts caused by muscle movements and blinks, resulting in approximately 5% of 100ms intervals of EMG data and trials being excluded from the analysis. Less than 2% of trials were excluded from the analyses using the standard deviation (SD) method (Wilcox, 1992) with the criterion value standing at 3 SDs per muscle. Before statistical analysis, EMG data on each facial muscle were averaged over 18 trials with the same emotional expression for left-and right-wing politicians.

#### 2.3.3 Evaluative rating

To test whether politicians’ facial expressions described in the subject-verb sentences elicited evaluative judgments in the reader, we asked participants to rate each sentence on a liking scale. Liking ratings of subject-verb sentences were provided after stimulus presentation by right-hand clicking on a 5-point Likert scale from 1 (*I don’t like it at all*) to 5 (*I like it very much*).

#### 2.3.4 Emotions towards target politicians

Participants’ emotional attitudes towards target politicians were assessed through a PANAS scale consisting of 3 positive (joy; enthusiasm; excitement) and 3 negative (anger; sadness; disappointment) emotions. The respondents indicated the degree to which thinking about the target politician elicited the given emotion on a 7-point Likert scale from 1 (*not at all*) to 7 (*very much*).

#### 2.3.5 Voting intention towards target politicians

To assess participants’ voting intentions we asked them to report on the likelihood of their voting for the target politicians in the coming elections on a 7-point Likert scale from 1 (*not at all likely*) to 7 (*very likely*).

## 3. Results

Electrophysiological and behavioral data were inspected for normality and then analyzed using a series of repeated measure analysis of variance (ANOVA) and correlational analysis, as detailed below. Post-hoc analysis was conducted using Bonferroni correction for multiple comparisons. Partial eta squared (η_p_^2^) was computed as a measure of effect size for significant main effects and interactions. By convention, η_p_^2^ effect sizes of ~ .01, ~.06, and ~.14 are considered small, medium and large, respectively [56].

### 3.1 Manipulation check

All participants reported being left-wing (*M* = 1.96, *SD* = 0.56). Alfano (*M* = 5.24; *SD* = 1.02) and Berlusconi (*M* = 5.65; *SD* = 0.93), were considered as representing the right-wing, whereas Bersani (M = 4.92; SD = 1.16) and Renzi (M = 4.80; SD = 1.20) were considered as representative of the left-wing, respectively. Repeated measures ANOVA showed that positive emotion verbs were considered as more positive (M = 5.68; SD = 0.59) than negative emotion verbs (M = 2.52; SD = 0.56), (*F*_1,28_ = 334.901, *p* < .0001, η_p_^2^ = .923).

### 3.2 Facial muscle activation pattern in response to ingroup and outgroup politicians’ expressions

EMG data were analyzed using a 2 (Political orientation: left-wing/ingroup, right-wing/outgroup) × 2 (Facial expression: smile, frown) × 6 (Time: 1–6 bins of 500ms) repeated measures ANOVA for the ZM, OO and CS muscles separately.

#### 3.2.1 Zygomaticus Major (ZM)

**Fig 1:**
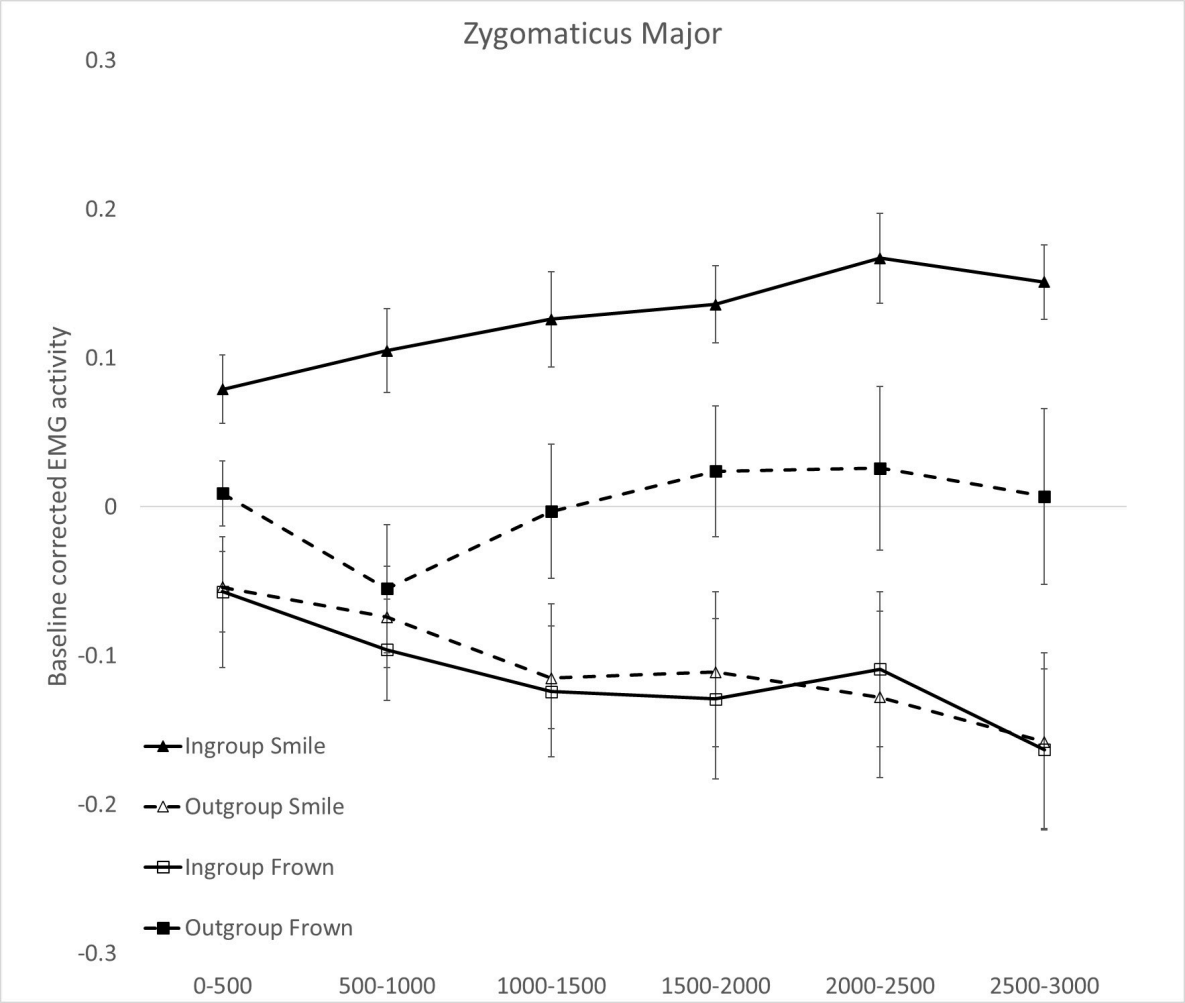
Zygomaticus Major muscle activity for left-wing versus right-wing politicians’ verbal expressions of smiles (panel A) and frowns (panel B). Responses are broken down in six 500ms-time windows of stimulus exposure (0 to 500ms; 500 to 1,000ms; 1,000 to 1,500ms; 1,500 to 2,000ms; 2,000 to 2,500ms; 2,5000 to 3,000ms). Error bars indicate standard errors of the mean.

The Political orientation × Facial expression × Time ANOVA on ZM data showed a main effect of Political orientation (*F*_1,28_ = 5.877, *p* = .022, η_p_^2^ = .173). Facial muscle activation was higher for ingroup (*M* = .013, *SE* = .02), compared to outgroup politician’s expressions (*M* = -.046, *SE* = .03). The two-way Political orientation × Facial expression interaction was significant (*F*_1,28_ = 11.712, *p* = .002, η_p_^2^ = .295), which was further qualified by the significant Political orientation × Facial expression × Time interaction (*F*_5,140_ = 7.501, *p* = .002, η_p_^2^ = .211). In line with the hypothesis of an enhanced smile response to smile depictions of ingroup but not outgroup politicians, Fig 1 shows a consistent and gradual increase in ZM activation when participants read about smile expressions of ingroup politicians, and opposite trends when participants read about ingroup frown expressions or outgroup smiles, with modulation peaking in the last time bins of the examined time window (2000-3000ms). Pairwise comparisons (based on Bonferroni tests) revealed that ZM activation was significantly higher when participants read about smile expressions attributed to ingroup compared to outgroup politicians across all time bins (0 – 3000ms; all *p*s < .011; Fig 1). Moreover, while reading frown expressions, there was a suppression of ZM activity for frowns attributed to ingroup compared to outgroup politicians (Fig 1), with significant differences emerging only at bin 1 (p = .033), and bin 6 (p = .035). Additionally, when participants were presented with smile and frown expressions of ingroup politicians their ZM activation was significantly higher for the former compared to the latter across all time bins (all *p*s < .002). Instead, there was no consistent difference in ZM activation for smile and frown expressions of outgroup politicians (all *p*s>.08). In sum, participants responded with an enhanced smile at the ZM level for ingroup compared to outgroup politicians’ smile depictions.

#### 3.2.2 Orbicularis Oculi (OO)

The Political orientation × Facial expression × Time ANOVA on OO data showed a main effect of Political orientation (*F*_1,28_ = 15.228, *p* = .001, η_p_^2^ = .352) with higher EMG activation for ingroup (*M* = .110, *SE* = .02) compared to outgroup politician’s expressions (*M* = -.034, *SE* = .04); a main effect of Facial expression (*F*_1,28_ = 10.803, *p* = .003, η_p_^2^ = .278) with higher EMG for positive (*M* = .107, *SE* = .02) compared to negative (*M* = -.031, *SE* = .04) facial expressions; and a main effect of Time (*F*_5,140_ = 17.205, *p* < .0001, η_p_^2^ = .381).

**Fig 2:**
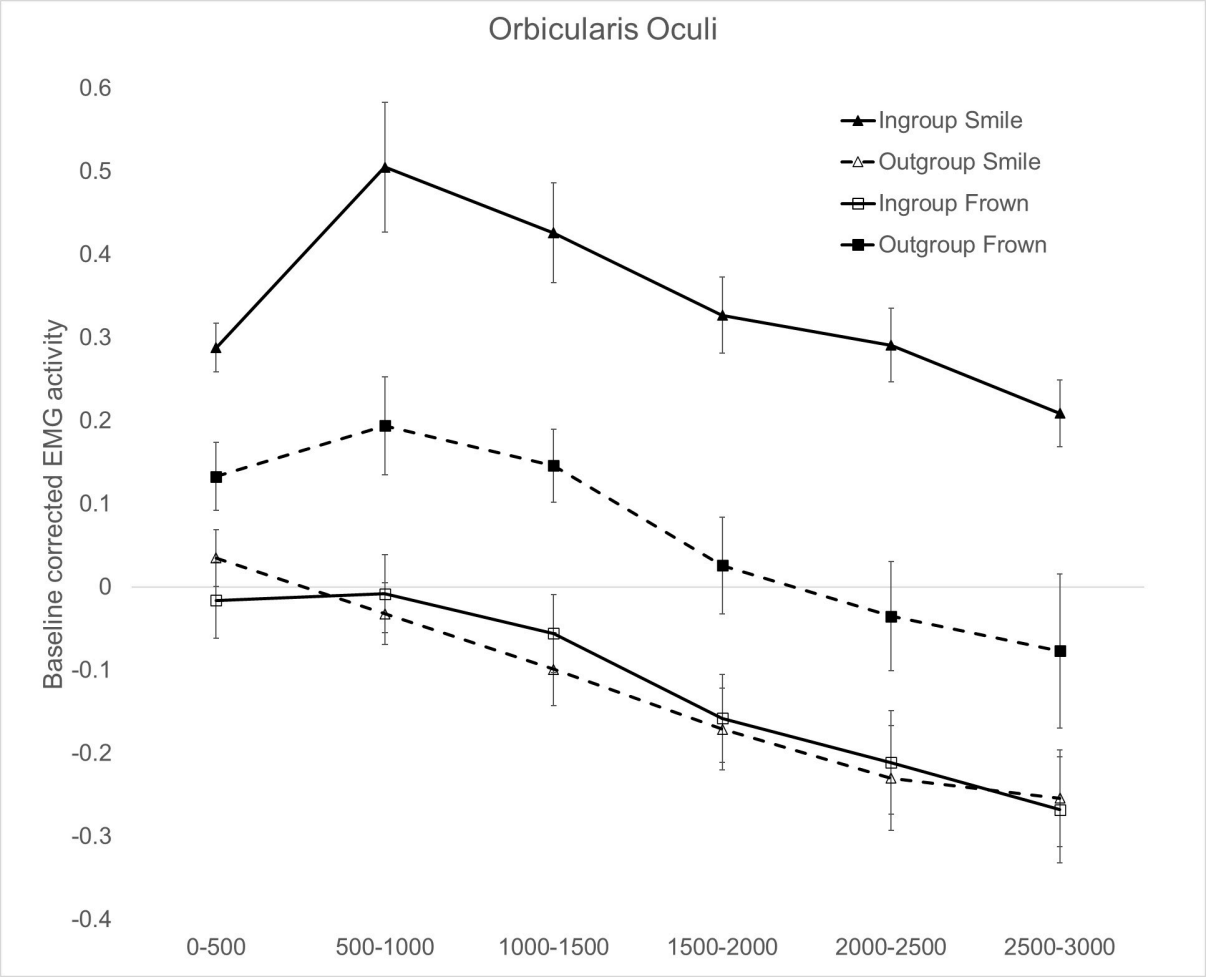
Orbicularis oculi muscle activity for left-wing versus right-wing politicians’ verbal expressions of smiles (panel A) and frowns (panel B). Responses are broken down in six 500ms-time windows of stimulus exposure (0 to 500ms; 500 to 1,000ms; 1,000 to 1,500ms; 1,500 to 2,000ms; 2,000 to 2,500ms; 2,5000 to 3,000ms). Error bars indicate standard errors of the mean.

The Political orientation × Facial expression interaction was also significant (*F*_1,28_ = 46.925, *p* < .0001, η_p_^2^ = .635) and it was further qualified by the significant Political orientation × Facial expression × Time interaction (*F*_5,140_ = 6.577, *p* < .0001, η_p_^2^ = .196). In line with our expectation of a more genuine smile in response to ingroup rather than outgroup leaders’ expressions, results show a robust OO activation while participants were reading about smile expressions attributed to ingroup politicians with an early peak around the second time bin (500-1000ms). Conversely, a comparably weaker early OO activation was observed while participants were reading about outgroup frown expressions (Fig 2). Pairwise comparisons (based on Bonferroni tests) revealed that participants reacted with a higher OO activation when reading of smile expressions attributed to ingroup compared to outgroup politicians, across all time bins (0–3000ms upon stimulus onset; all *p*s < .0001; Fig 2). When participants were presented with frowns, OO activation was significantly suppressed for ingroup compared to outgroup politicians’ expressions, across time bins (all *p*s < .010; Fig 2). In addition, smiles compared to frown expressions of ingroup politicians were associated with greater OO muscle activation in all time bins (all *p*s < .0001), whereas the inverse pattern was observed for expressions of outgroup politicians: smiles induced significantly lower activation of the OO muscle compared to frowns, across all time bins (all *p*s < .050). Taken altogether, these results offer support to the hypothesis of a nuanced smile response, indicating more positive affect when participants were exposed to smile depiction of ingroup compared to outgroup politicians.

#### 3.2.3 Corrugator Supercili (CS)

The Political orientation × Facial expression × Time ANOVA on EMG data recorded on the CS showed a main effect of Political orientation (*F*_1,28_ = 30.159, *p* < .0001, η_p_^2^ = .519), with higher CS activity for ingroup (*M* = .137, *SE* = .03) compared to outgroup politician’s expressions (*M* = -.014, *SE* = .02); a main effect of Facial expression (*F*_1,28_ = 13.829, *p* = .001, η_p_^2^ = .331), with higher activation for negative (*M* = .135, *SE* = 0.03) compared to positive (*M* = -.012, *SE* = 0.03) facial expressions; and a main effect of Time (*F*_5,140_ = 13.864, *p* = .019, η_p_^2^ = .121), with higher CS activation during bin 1 (0-500ms, M = .105, SE = .02) and bin 2 (500–1000ms, M = .117, SE = .03) compared to bin 3–6 (1000-3000ms, M = .063–0.14, SE = .03-.04; all *p*s < .030).

**Fig 3:**
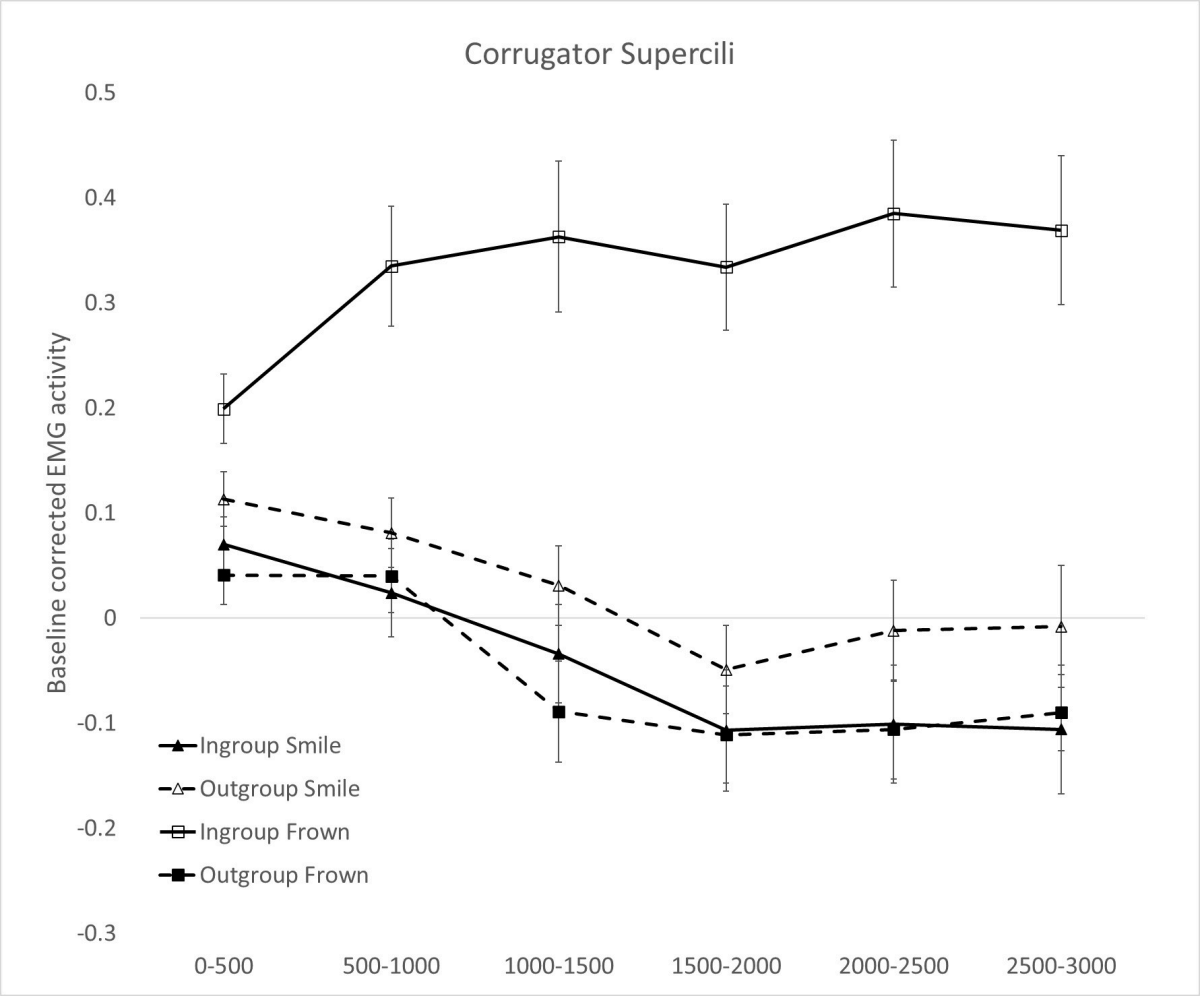
Corrugator Supercilii muscle activity for left-wing versus right-wing politicians’ verbal expressions of frowns (panel A) and smiles (panel B). Responses are broken down in six 500ms-time windows of stimulus exposure (0 to 500ms; 500 to 1,000ms; 1,000 to 1,500ms; 1,500 to 2,000ms; 2,000 to 2,500ms; 2,5000 to 3,000ms). Error bars indicate standard errors of the mean.

The Political orientation × Facial expression interaction (*F*_1,28_ = 41.138, *p* < .0001, η_p_^2^ = .595) was also significant which was further qualified by the significant Political orientation × Facial expression × Time interaction (*F*_5,140_ = 10.937, *p* < .0001, η_p_^2^ = .281). Fig 3 shows strong sustained CS activity when reading frown expressions of ingroup politicians with activation peaks at bin 5 (2000-2500ms) and a weaker late deactivation when reading about smile expressions of ingroup politicians. Pairwise comparisons (based on Bonferroni tests) revealed that the activation of the CS was higher when participants read about frown expressions of ingroup compared to outgroup politicians across all time bins (all *p*s < .0001; Fig 3). In terms of smile expressions, a greater suppression of the CS activation was evidenced for expressions of ingroup compared to outgroup politicians (Fig 3). When participants were presented with expressions of ingroup expressions their CS response was higher for frowns compared to smiles in all time bins (all *p*s < .0001). Instead, the reverse pattern was observed when they were reading expressions of outgroup politicians: CS activation was lower for frown compared to smile expressions, with significant differences emerging at bin 1 (0-500ms, p = .030) and bin 3 (1000-1500ms, p = .006). In sum, findings on the frown muscle activation (CS) corroborate the activation pattern found for smile facial muscles of ZM and OO indicating a more enhanced and emotion-congruent facial response when reading about expressions of ingroup compared to outgroup politicians.

### 3.3 Evaluative ratings

To test whether participants liked to a different extent the facial expressions displayed by politicians of their own compared to the opposite political orientation, self-report data were analyzed in a 2 (Facial expression: smile, frown) × 4 (Politicians: Alfano, Berlusconi, Bersani, Renzi) repeated measures ANOVA. Results revealed the main effects of Politician, (*F*_3,84_ = 34.455, *p* < .0001, η_p_^2^ = .552) indicating higher liking rates for ingroup compared to outgroup politicians (see Table 1). This effect was further qualified by the significant interaction between Politician and Facial expression (*F*_3,84_ = 33.283, p < .0001, η_p_^2^ = .543). Pairwise comparisons (based on Bonferroni tests) showed that participants reported significantly higher liking rates for the smiling of ingroup compared to outgroup politicians Berlusconi (all *p*s < .001). Instead, similar rates of liking were reported for frowning of ingroup compared to outgroup politicians Berlusconi (all *p*s>.480).

**Table 1:** Mean (± SD) scores on liking of politician verbal expressions reported by participants for each target political leader.

		Liking of politician verbal expressions		
	Target Politicians	*All emotion expressions*	*Positive emotion expressions*	*Negative emotion expressions*
Left-Wing (ingroup)	Bersani	3.02 ± 0.08	3.46 ± 0.71	2.57 ± 0.45
	Renzi	2.78 ± 0.07	3.37 ± 0.71	2.53 ± 0.36
Right-Wing *(outgroup)*	Alfano	2.26 ± 0.10	1.95 ± 0.67	2.61 ± 0.72
	Berlusconi	2.17 ± 0.11	1.73 ± 0.88	2.65 ± 0.86

### 3.4 Emotions toward target politicians

**Fig 4:**
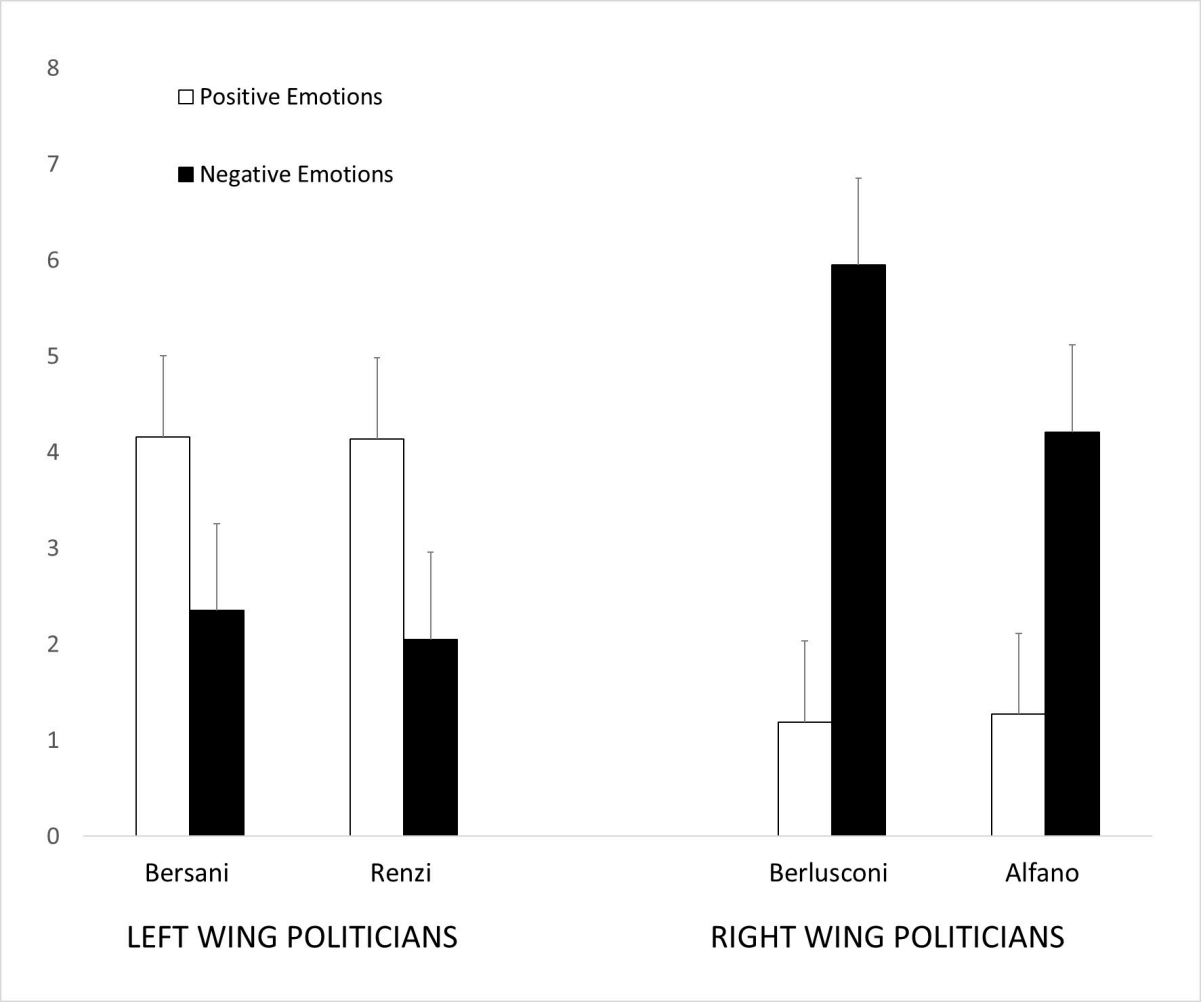
Positive and negative emotions reported for ingroup (left-wing) and outgroup (right-wing) target politicians. Error bars indicate standard errors of the mean. * *p <* .05. ** *p <* .01. *** *p <* .001.

Affective responses were analyzed into a 2 (Emotions: positive, negative) × 4 (Politician: Alfano, Berlusconi, Bersani, Renzi) repeated measures ANOVA. The analysis showed a main effect of Emotion (*F*_1,19_ = 28.045, *p* < .0001, η_p_^2^ = .596) showing higher scores for negative (*M* = 3.64, *SE* = .14) relative to positive emotions (*M* = 2.69, *SE* = .11); a main effect of Politician, (*F*(3, 57) = 9.864, *p* < .0001, η_p_^2^ = .342) showing different scores across target politicians; and a significant interaction between Emotion and Politician (*F*_3,57_ = 102.085, *p* < .0001, η_p_^2^ = .843; Fig 4). Pairwise comparisons (based on Bonferroni tests) showed that participants reported more positive emotions towards ingroup politicians Bersani (*M* = 4.16, *SD* = 1.03) and Renzi (*M* = 4.14, *SD* = 1.55) compared to outgroup politicians Berlusconi (*M* = 1.19, *SD* = 0.40; all *p*s < .001) and Alfano (*M* = 1.27, *SD* = 0.38; all *p*s < .001). Additionally, participants reported more negative emotions towards outgroup politicians Berlusconi (*M* = 5.95, *SD* = 1.03) and Alfano (*M* = 4.21, *SD* = 0.96) compared to ingroup politicians Bersani (*M* = 2.35, *SD* = 0.97; all *p*s < .001) and Renzi (*M* = 2.05; *SD* = .99; all *p*s < .001).

### 3.5 Voting intention towards target politicians

The ANOVA performed on voting intentions ratings showed that participants would most likely vote for ingroup politicians Bersani (M = 5.10, SE = .34) and Renzi (M = 4.63, SE = .30), compared to outgroup politicians Alfano (M = 1.05, SE = .05) and Berlusconi (M = 1.15, SE = .07) in the upcoming elections (*F*_3,84_ = 91.960, *p* < .001, η_p_^2^ = .767).

### 3.6 Relationship between facial responses, evaluative judgements, emotions and voting intentions towards political leaders

Finally, we examined correlations between facial EMG responses to ingroup and outgroup facial expressions, evaluative judgments, reported emotions and voting intentions towards ingroup and outgroup politicians. We did not find significant correlations between EMG and behavioral data.

## 4. Discussion

Politicians may strategically use smiles to influence their voters’ behavior [13, 14, 57]. However, a critical question emerges: does reading about a politicians’ smile spontaneously evoke nuanced smile reactions, and do these reactions differ depending on whether readers share the same or opposing political orientation? In the present study we examined facial EMG activity to test whether reading about ingroup or outgroup politicians smiling would elicit morphologically different smiles in the faces of readers. As expected, we found that participants facially responded with a smile pattern involving the activation of both lip puller (ZM) and eye corner (OO) facial muscles to phrases describing ingroup but not outgroup politicians smiling. By going beyond a one-size fits-all approach to detecting smiles, and by examining the temporal dynamic of facial muscle activation we were able to uncover a more nuanced smile reaction in our participants, unveiling fine-grained differences in early responses to ingroup compared to outgroup politicians’ smiling portrayals. This is the first study to show that a smile pattern which is underpinned by the activation of both ZM and OO facial muscles, can be observed not just when one sees a loved political leader smiling, but also when reading about their smiling expressions in written media. Interestingly while in all muscles we were able to detect emotionally congruent reactions to ingroup politicians’ expressions (i.e., ZM and OO activation for ingroup smiles and CS activation for ingroup frowns), the OO muscle showed the earliest peak of activation and appeared particularly sensitive to the ingroup vs. outgroup manipulation as compared to the other muscles.

These findings importantly extend previous research based on exposure to visual [2, 51] or auditory cues [7] as they are the first to demonstrate that by stringently controlling the perceptual features of target’s smile through the use of linguistic material, mere conceptual knowledge about the target can finely modulate the readers’ facial response. This nuanced modulation is evident in the differential recruitment of readers’ facial muscles when they engage with verbal descriptions of smiles of ingroup and outgroup politicians [15]. These results expand upon previous work by Fino et al., [29] revealing fine-grain distinctions in the readers’ smile response, particularly when tracking concurrent activation of distinct facial muscles. Not all smiles are equal they say, and when our participants were reading of ingroup politicians smiling, spontaneous muscle contractions could be captured in the eye corner (OO) and the lip puller (ZM) muscles, suggesting a Duchenne (aka positive affect) smile reaction for ingroup but not outgroup politicians’ smiles [4, 17]. This was confirmed by the reported emotions towards target politicians which were significantly more positive for ingroup and more negative for outgroup political leaders, consistent with our predictions.

By emphasizing the importance of the eye region in discerning highly nuanced smile reactions [15], our study further extends previous research focusing on the Italian political context highlighting the automatic attention-grabbing effect of a politician’s eyes on voters. In their study, Liuzza, and colleagues [58] examined how the eye gaze of a prominent right-wing Italian politician (Berlusconi) had a strong catching power over the attention of potential voters, enhancing gaze following behavior of supporters and inhibiting that of opponents. By using a different experimental paradigm, in the present study we measured reflexive smile patterns emerging very rapidly in the eye corner area of supporters, demonstrating a highly differentiated smile response to ingroup but not outgroup smile displays. What is remarkable is that such fine-grained differences in supporters’ smile reactions were observed in facial responses to language portraying politicians smiling, going beyond previous evidence on the social and political factors affecting such responses. The significance of language as a means of exerting influence to achieve individual, social and political goals has been widely demonstrated [1, 59]. Findings of our study take a significant step forward in enhancing our understanding of how language can sensibly shape subtle behaviors. The high facial sensitivity to language describing politicians’ emotionally embedded behavior, underscores the embodied nature of social cognitions highlighting how language-driven facial effects are influenced by social information. These observations carry substantial implications for eliciting congruent emotions and social judgments on political leaders, potentially influencing voting behavior. That is, reading about politicians in newspapers or in social media can have the power to affect voters’ behavior much in the same way as when exposed to images of political leaders. Political campaigns often emphasize nonverbal aspects of politicians’ behavior, such as smile expressions or skin complexion, which can be visually manipulated in ways that are difficult to detect and profoundly influence how voters perceive and feel about political candidates [60]. In a complementary approach, we turn our attention to language, demonstrating its comparable efficacy in mobilizing support beyond conscious means and through embodied responses. Our findings seem to suggest that measuring fleeting facial reactions while being exposed to written reports on political candidates’ emotional behaviors could serve as an implicit index of embodied political attitudes with potential implications for predicting voting decisions [61]. This holds particular significance for opinion polls, which rely predominantly on self-report data and are consequently constrained by controlled factors.

The observation that the facial reaction pattern underpinned by the activation of both ZM and OO muscles—which reflects the signature of Duchenne smiles–was evidenced only for linguistic portrayals of ingroup but not outgroup politicians’ smiles suggests distinct processing mechanisms for expressions of ingroup and outgroup leaders. This is consistent with research on social modulation of facial EMG response demonstrating that emotionally congruent facial reactions are tendentially more enhanced for ingroup members, people we like and those towards whom we harbor affiliative intentions [12, 62]. Our findings also converge with evidence showing that Duchenne smiles are associated with greater liking and perceived psychological proximity [63], which is expectedly higher towards ingroup compared to outgroup members. In line with established research lines on intergroup perception [64–66], people show positive bias towards ingroup members as compared to outgroup members. Consequently, they are more inclined to judge an ingroup smile as genuine, authentic and indicative of positive feelings or intentions. Conversely, in the case of outgroup smiles, it is more probable that the perceivers believe that the smile masks a feeling of superiority, a desire to manipulate or lie, or simply negative affect, particularly in the political context [16]. In absence of more contextual information, it is plausible that while reading about ingroup and outgroup politicians’ smiles participants may have made inferences about smiles meaning and/or genuineness, which may have been reflected in their different smile response. This interpretation is consistent with the pattern of reported liking rates by our participants which were significantly higher for ingroup and lower for outgroup politicians’ smiles. As suggested by previous research, smiles that are perceived as more authentic and truer are liked more and elicit more positive emotions compared to smiles that are perceived as fake or as expressing superiority, which are associated with more negative emotions [4, 15, 16, 22]. Although it has been noted that Duchenne smiles can also be displayed deliberately and in absence of positive affect [23], the fact that we measured spontaneous facial reactions while participants were unaware that their facial muscle activity was being recorded speaks against the possibility that they may have intentionally smiled with their eyes.

The present research goes beyond the one-size-fits-all approach to smile responses which typically considers ZM activation as the main and only index of smile reactions. Indeed, we found that not only the ZM but also the OO muscle appeared consistently engaged in the task and sensitive to our language manipulations showing the highest and earliest peaks of EMG activations relative to the other muscles. Through the combined measurement of more facial muscle activity parameters, based on the evidence on smile variability [15, 17], and the analysis of the temporal dynamic of facial muscle activation we were able to reach a more in-depth analysis of differences in facial reactions, which may have been previously masked by measurement approaches employed in other studies.

The significant ZM and OO activation (reflecting the Duchenne marker) exclusively in response to ingroup but not outgroup smiles, challenges the existing interpretation of an intriguing empirical evidence in this area, namely that smiles are sometimes indiscriminately corresponded across social groups and contexts [10]. While smiles are often used to smooth social interactions (i.e., affiliative smiles), previous research may have neglected more subtle differences in smile reactions. In relation to politicians’ smiles, the only previous study to concurrently assess responses at the ZM and OO muscles found no evidence for increased activity in OO for ingroup relative to outgroup politicians [51]. However, it is worth noting that this study employed ecological dynamic stimuli and assessed facial reactions over an extended 13-s time frame. The question of whether stimuli related features or timeframe exposure may have influenced results cannot be completely ruled out. Activation of the OO might be sensitive to post-stimulus onset time with activity peaking in earlier windows and diminishing over time as demonstrated by previous research [27]. Hence, emphasizing the importance of a temporal dynamics analysis becomes critical as it has the potential to reveal more subtle differences in facial muscle activation unfolding over time.

The evidence of the present study fully supports the hypothesis of an enhanced response for ingroup compared to outgroup emotion expressions among left-wing participants. This may seem at odds with results of Fino et al., [29], which revealed a similar ZM activation for both ingroup and outgroup politicians’ smiles among left-wing voters. The latter study also observed left-wing participants displaying smiles in response to outgroup anger expressions, indicative of *schadenfreude*, or pleasure at the misfortune of others. The differences in facial reaction patterns may be due to the linguistic stimuli employed in both studies, which varied in terms of linguistic abstraction. While the present study employed concrete verbs referring directly to facial behaviors of politicians “Berlusconi smiles/frowns”, stimuli in Fino et al., [29] employed both concrete and abstract verbs referring to a higher extent to emotional states of political leaders “Bersani is happy/angry”. Research on linguistic abstraction [67] shows that abstract verbs carry a higher emotional charge. Hence, left-wing participants in Fino et al., [29] might have been more profoundly influenced by the level of abstraction of the linguistic stimulus, which attributed emotional states to the protagonist of the behavior.

However, our research is not without limitations. On the one hand, findings fully corroborated the hypotheses through an experimental procedure that involved a substantial number of repeated measurements of psychophysiological (EMG) reactions, ensuring sufficient power for statistical analysis. On the other hand, the relatively small number of participants predominantly comprising female left-wingers represents a potential drawback in terms of generalizability of results. Previous literature suggests that there may be important differences in the way left-and right-wing voters react to in/outgroup political leaders, which can be explained by differences grounded in motivational and socio-cognitive processes. For instance, right-wing individuals tend to have higher preference for stability, certainty, cognitive closure (for a meta-analysis see [68]). Hence, it could be expected that right-wing/conservative voters would be more inclined to facially react with an enhanced and emotionally congruent facial response to ingroup political leaders compared to left-wing/liberal voters. Although there is some support for this hypothesis [29], additional research is necessary to investigate this issue.

Furthermore, our sample was predominantly female with target politicians examined being all male, preventing an exploration of gender related effects. Research shows that compared to males, females are more emotionally expressive and exhibit a higher facial responsivity to others’ emotion expressions [69]. Furthermore, recent studies have shown that female politicians display more intense and affiliative type of smiles compared to their male counterparts and are judged more negatively if they do not [70]. The existing body of EMG research predominantly focuses on emotion expressions of male politicians and does not extensively examine facial reactions of both male and female supporters/opponents (except for [2]). This represents a gap in the current literature offering a promising avenue for future research. Future studies should also examine the joint effects of gender and political ideology across both leaders and potential voters, exploring their impact on modulating facial reactivity to ingroup and outgroup emotion expressions. Additionally, we did not provide information about the interaction context in which politicians’ smiles were displayed, whether they were in response to a praising comment (i.e., enjoyment smile) or an angry opponent’s remark (i.e., dominant smile). Future studies should address this issue and consider a more stringent manipulation of the meaning of the smiles presented through verbal material. For instance, presenting phrases such as “Politician smiles joyfully” vs. “Politician smiles politely” or with an air of superiority, may be a useful approach to examine fine grain differences in supporters’ and opponents’ smile reaction patterns.

Despite these limitations our findings are the first to highlight how language and social information can shape embodied mechanisms underlying facial reactivity, which can be of relevance particularly to the field of social and political communication. They also offer evidence suggesting that existing discrepancies in empirical findings on facial reactions to smiles in the intergroup context could potentially be reconciled by going beyond one-size-fits-all approaches that rely on assessment of ZM activity only. This has implications for future research aimed at unravelling the complexities of facial response to smiles, whether presented conceptually or not, in different social and emotional contexts. The integration of more facial markers involved in different smile expressions would also allow to capture more complex and nuanced smile patterns. Moreover, using linguistic portrayals of others’ smiling offers a useful approach in terms of more stringently testing social interaction and contextual factors. Also combining additional neuro/psycho-physiological indicators of emotional states while facially responding to different types of smiles, as suggested by recent research [17, 18], may provide a more comprehensive picture of the processes behind different smile reactions, in different social and emotional contexts. This approach has the potential to overcome possible biases inherent in research relying solely on behavioral and self-report measures.

## 5. Conclusions

Research in embodied cognition has demonstrated the intricate connections between language and our bodily experience highlighting that words can deeply resonate with us and may get under our skin in ways that are becoming increasingly clear, as they are variegated and complex [6, 28–30, 71]. However, the extent to which they are able to do so relies on the larger social context and is sensibly modulated by social factors. The identification of markedly distinct smile reactions to linguistic portrayals of ingroup and outgroup politicians’ expressions adds another layer to our understanding of how language and social information can influence embodied simulation in a highly nuanced fashion.

## Supporting information

S1 File (XLSX)
